# Mastocytosis—A Review of Disease Spectrum with Imaging Correlation

**DOI:** 10.3390/cancers13205102

**Published:** 2021-10-12

**Authors:** Ahmed Elsaiey, Hagar S. Mahmoud, Corey T. Jensen, Sergio Klimkowski, Ahmed Taher, Humaira Chaudhry, Ajaykumar C. Morani, Vincenzo K. Wong, Usama I. Salem, Sarah M. Palmquist, Khaled M. Elsayes

**Affiliations:** 1 Houston Methodist Hospital, Houston, TX 77030, USA; aelsaiey@houstonmethodist.org; 2 Department of Diagnostic Radiology, Yale New Haven Health at 1939 Bridgeport Hospital, Bridgeport, CT 06610, USA; hag.smahmoud@gmail.com; 3 MD Anderson Cancer Center, Department of Diagnostic Imaging, University of Texas, Houston, TX 77030, USA; CJensen@mdanderson.org (C.T.J.); SPKlimkowski@mdanderson.org (S.K.); AMorani@mdanderson.org (A.C.M.); vkwong@mdanderson.org (V.K.W.); USalem@mdanderson.org (U.I.S.); smoorhead@mdanderson.org (S.M.P.); 4 Transitional Year Residency Program, Trinity Health Midatlantic, Nazareth Hospital, Philadelphia, PA 19152, USA; artahermd@gmail.com; 5 Department of Radiology, The State University of New Jersey, Piscataway, NJ 08854, USA; chaudhhu@njms.rutgers.edu

**Keywords:** systemic mastocytosis, cutaneous mastocytosis, mast cell tumor, imaging review

## Abstract

**Simple Summary:**

In this review will discuss the clinical presentation, pathophysiology, and role of imaging in detection and extent estimation of the systemic involvement of the disease, in addition to demonstration of appearance on varying imaging modalities. Familiarity with the potential imaging findings associated with mastocytosis can aid in early disease diagnosis and classification and accordingly can lead directing further work up and better management.

**Abstract:**

Mastocytosis is a rare disorder due to the abnormal proliferation of clonal mast cells. Mast cells exist in most tissues, mature in situ from hematopoietic stem cells and develop unique characteristics of local effector cells. Mastocytosis develops by activation mutation of the KIT surface receptor which is involved in the proliferation of a number of cell lines such as mast cells, germ cells, melanocytes, and hematopoietic cells. It manifests as two main categories: cutaneous mastocytosis and systemic mastocytosis. Imaging can play an important role in detection and characterization of the disease manifestation, not only by radiography and bone scans, but also magnetic resonance imaging and computed tomography, which can be more sensitive in the assessment of distinctive disease patterns. Radiologists should be aware of various appearances of this disease to better facilitate diagnosis and patient management. Accordingly, this review will discuss the clinical presentation, pathophysiology, and role of imaging in detection and extent estimation of the systemic involvement of the disease, in addition to demonstration of appearance on varying imaging modalities. Familiarity with the potential imaging findings associated with mastocytosis can aid in early disease diagnosis and classification and accordingly can lead directing further work up and better management.

## 1. Introduction

Mastocytosis is an uncommon disorder with an estimated prevalence of 1 in 10,000 persons characterized by abnormal proliferation and accumulation of clonal mast cells [1]. Organ involvement and clinical features further classify the disease as cutaneous mastocytosis (CM) and systemic mastocytosis (SM). First case reports of this disorder date back to 1869 descriptions of cutaneous mastocytosis by Nettleship and Tay. Subsequently in 1936, Sezary described the first case of mastocytosis not limited to the skin and involving other organs [2]. Tumorgenesis begins with a somatic mutation in the tyrosine kinase receptor, c-Kit, which results in uncontrolled stimulation of its ligand, stem cell factor (SCF). Herein, we describe the pathophysiology of mastocytosis, the most typical clinical presentations, and imaging correlation.

## 2. Pathophysiology

Mast cells are an integral component of the immune system and are present in most tissues. Mast cells play a crucial role in the regulation of physiological functions, including vasodilation, angiogenesis, and regulation of many cell types such as macrophages, T cells, B cells, and eosinophils [3]. In addition to regulatory functions, mast cells are also capable of immobilizing and killing microorgranisms and parasites [4]. There are two mast cell types: mast cells containing tryptase (MCT) and mast cells containing tryptase and chymase (MCTC) [5,6]. Both types are interconvertible under certain conditions [7].

Mast cell progenitors are derived from hematopoietic stem cells in the bone marrow. After mobilization to blood and ultimately tissue, full differentiation and maturation occur [8,9]. This differentiation occurs when the stem cell factor (SCF), a progenitor’s growth factor, binds its receptor on mast cells, KIT (CD117) [9,10]. Unlike other hematopoietic cells, mast cells express many KIT receptors that remain responsive to SCF, so mutations in KIT or dysregulation of SCF lead to uncontrolled mast cell proliferation, and mastocytosis occurs [9]. 

The *KIT* gene is located on chromosome 4 and encodes a transmembrane tyrosine kinase receptor responsible for mast cell proliferation and survival. The extracellular domain of the KIT gene has two important sites: a binding site for SCF and a dimerization site. The cytoplasmic portion comprises the juxtamembrane domain and tyrosine kinase domain which is the site for receptor phosphorylation. When SCF binds the receptor, autophosphorylation of tyrosine kinase occurs and facilitates the transduction process [11,12]. 

A proliferating somatic mutation of KIT is the most common molecular defect in mastocytosis. It affects the juxtamembrane domain of the KIT receptor and hinders the regular kinase activity resulting in abnormal mast cell survival and tissue accumulation. The most common mutation, D816V, affects the phosphorylation site of the KIT tyrosine kinase receptor and results in activation of the mast cell [9,13]. This mutation also leads to abnormal activation of signal transducer and activator transcription 5 (STAT5), a transcription factor essential for proliferation of mast cells. The STAT5/PI3K/AKT pathway is responsible for the survival of abnormal mast cells and the development of mastocytosis [11]. Other less common mechanisms of disease can include increased expression of SCF, aberrant interleukin 5 (IL-5) receptors, and genetic mutations in the TET2, SRSF2, and RAS genes.

Mast cells release mediators upon activation which are responsible for allergic inflammatory reactions including anaphylaxis. The pathologic accumulation of mast cells, mediator release, and the body’s response to those mediators lead to manifestations of mastocytosis. Mast cells have also been implicated in the inflammatory mechanism of oral diseases such as periodontitis [14]. The neurohormonal mediators interleukin-33 (IL-33) and Substance P (SP) stimulate mast cells and have been suggested in the pathophysiology of nasal polyposis [15].

## 3. Clinical Presentation and Diagnostic Criteria

Mastocytosis affects both children and adults with a variable and often complex symptom profile. The wide range of clinical presentations are based on organ involvement (local versus systemic) and disease course (indolent versus aggressive). Symptoms may be non-specific such as nausea and headache, and they can range in severity to include severe cardiac and neurologic manifestations or simple skin urticaria. Many of the symptoms correlate with the allergic mediators secreted by the clonal mast cells, such as histamine, heparin, cytokines, and leukotrienes.

## 4. Cutaneous Mastocytosis (CM)

Presentation of CM differs by age of the patient. Children almost exclusively have self-limiting disease localized to the skin, typically resolving by adolescence. CM can be associated with gastrointestinal cramping and risk of anaphylaxis. In contrast, adult-onset CM often has a systemic component and typically a chronic course [1,2].

CM is subclassified by the WHO into three main categories: urticaria pigmentosa (UP), diffuse cutaneous mastocytosis, and mastocytoma of the skin [16]. UP is the most common form of CM, and it presents as yellow to brown maculopapular lesions on the trunk and extremities, often sparing the face, palms, and soles. UP lesions can have a myriad of characteristics and differ based on age of the patient at diagnosis. They are polymorphic with various shapes and colors in children, and tend to be monomorphic in adults [12,16]. Polymorphic lesions are more prominent and usually fade with age. Monomorphic lesions in children frequently persist into adulthood. Other lesions associated with UP include petechiae, telangiectasia, hemorrhage, and blisters.

Diffuse CM is less common and seen in about 1–5% of children. It presents as generalized edema and erythema, possibly with hemorrhagic dermatitis and subsequent hyperpigmentation [17]. Another variant, mastocytoma, starts at an early age and rarely affects adults. It presents with a solitary macule or papule and may involve the palms and soles [16]. All types of CM are typically diagnosed by the presence of mast cells on skin biopsy [12]. Pediatric evaluation can forgo bone marrow biopsy in the absence of hepatosplenomegaly, peripheral blood cell abnormalities or lymphadenopathy [2].

## 5. Systemic Mastocytosis

SM typically presents in the 5th decade of life or later [18,19]. SM is subclassified by the WHO into four main categories: indolent SM, SM with an associated non-mast-cell lineage, aggressive SM, and mast cell leukemia [2]. SM results from the release of accumulated mast cells mediators in various organs [12]. Although extracutaneous, SM may be associated with skin lesions. Aggressive SM forms can frequently present without skin lesions. SM is characterized by heterogeneous clinical features such as pruritis, flushing, hypotension, GI symptoms, neuropsychiatric symptoms, musculoskeletal pain, and cardiac symptoms such as tachycardia and palpitations. [18].

In addition to laboratory and bone marrow findings, differentiating the various subtypes of SM requires evaluation of organ function and attention to WHO diagnostic criteria (Table 1 and Table 2) [20]. Organ involvement without dysfunction is associated with a better prognosis, whereas organ dysfunction related to massive mast cell infiltration is associated with an aggressive form of SM.

Indolent SM is the most common subtype. It indicates the presence of SM without any hematologic neoplasm, with very few B findings and no C findings (Table 1). Smoldering SM presents with two or more B findings and no C findings. Indolent SM portends a better prognosis than the smoldering variant. SM with an associated hematologic neoplasm is the second most common variant of SM. It meets the diagnostic criteria for SM along with a non-mast cell disorder such as myeloproliferative neoplasm (MPN), myelodysplastic syndrome (MDS), or acute myeloid leukemia (AML) [19,21]. The most common neoplasm associated with SM is chronic myelomonocytic leukemia (CMML) [22]. Rarely, SM is aggressive and presents with C finding(s). It has fewer mast cells in the bone marrow and is not associated with any hematologic neoplasm. However, it does result in massive infiltration of mast cells in various organs. [23]

Diagnosis of SM is dependent on the presence of major and minor WHO diagnostic criteria. Major criteria include the detection of mast cell infiltration in the bone marrow or other organ (Table 2). Minor criteria include atypical mast cells in the bone marrow biopsy, detection of KIT mutation at codon 816, mast cells exhibiting CD2/CD25, and baseline serum tryptase ≥ 20 ng/mL. Diagnosis of SM requires the presence of a major criteria with one minor criteria, or three minor criteria without any major criteria [20].

## 6. Imaging Findings

### 6.1. Skeletal

**Figure 1:**
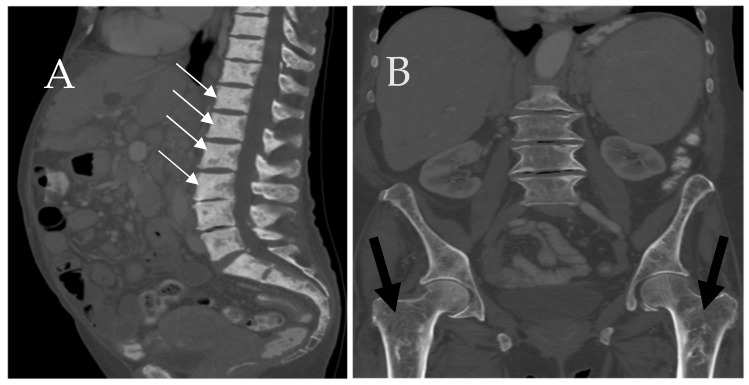
A 67-year-old female patient presenting with back pain. (**A**) Sagittal CT of the spine shows diffuse sclerosis of the axial skleton (white arrows). (**B**) Coronal CT shows a diffuse loss of normal bone trabeculations in bilalateral proximal femurs secondary to mast cell infiltrations (black arrows).

**Figure 2:**
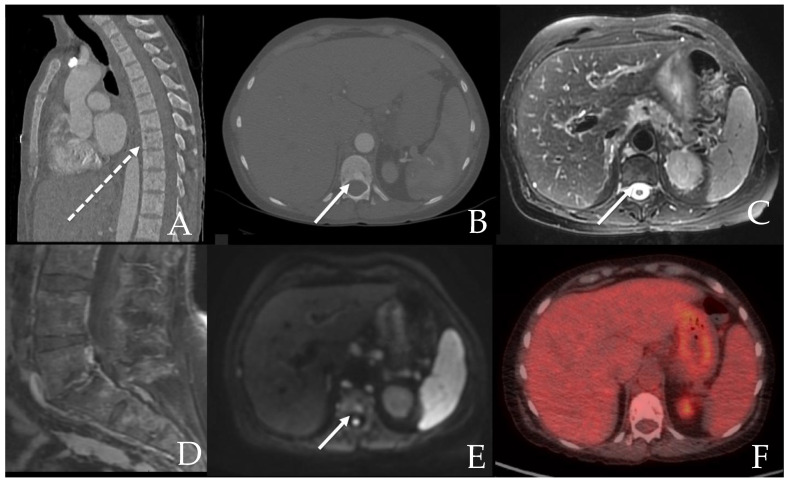
71 year old female with aggressive mastocytosis. Multimodality imaging demonstrates patchy sclerosis throughout the osseous structures on sagittal CT of the spine (**A**) with mild variable enhancement on post-contrast T1 weighted sagittal MRI of the spine (**D**). An example sclerotic lesion (arrow) on CT (**B**) is seen with mild T2 and DWI hyperintensity without PET hypermetabolism (**C**,**E**,**F**). A few lytic osseous foci (dashed arrow) were also noted (**A**).

The skeletal system is most commonly involved in SM and serves as a prognostic factor [24]. Osseous involvement is seen in up to 90% of SM patients. Magnetic resonance imaging (MRI) and computed tomography (CT) are the best imaging modalities to evaluate osseous lesions and bone marrow. CT is very helpful in evaluating for focal sclerotic or lytic lesions (Figure 1) [21]. Bone lesions in SM can be lytic, sclerotic, or mixed (Figure 2) [25,26]. Appearance of osseous involvement can be diffuse or focal. High tissue contrast with MRI makes this modality very useful in detecting bone marrow involvement [21]. Bone marrow involvement in SM appears hypointense on precontrast T1 weighted images with varying degrees of relatively mild enhancement and T2 weighted hyperintensity. Similar to other disease processes, diffusion weighted imaging can provide an assessment of lesion cellularity which may prove useful in following disease treatment (Figure 2) [27]. Fluorodeoxyglucose (FDG) positron emission tomography (PET) can demonstrate SM activity, but the exact role of this modality for SM is evolving [20].

### 6.2. GI Tract, Liver and Spleen

#### 6.2.1. GI Tract

**Figure 3:**
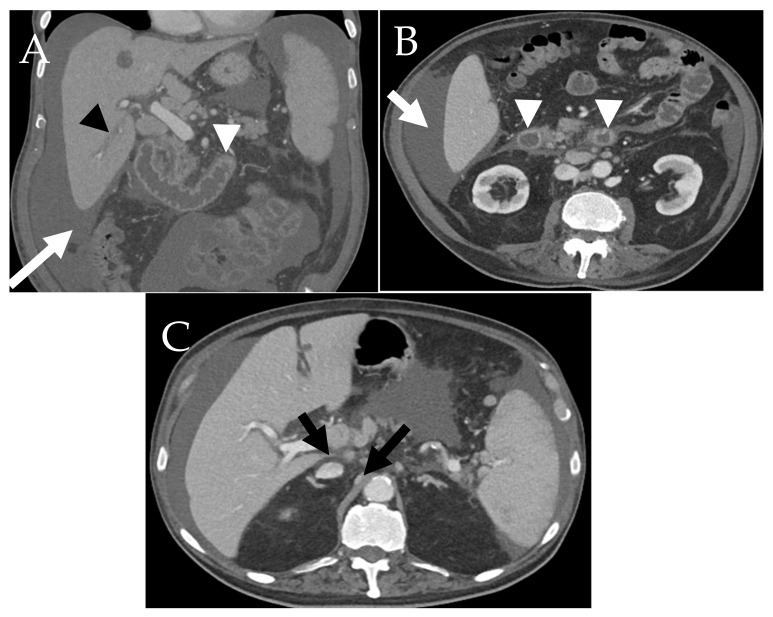
An 80-year-old male with a history of systemic mastocytosis with associated clonal hematological non-mast-cell lineage disease (SM-AHNMD) (**A**) Coronal contrast-enhanced CT and (**B**), (**C**) Axial contrast-enhanced CT scans demonstrate hepatomegaly, periportal edema (black arrowhead), moderate ascites (white arrow), peri-portal and retroperitoneal lymphadenopathy (black arrows), and duodenal and jejunal wall thickening (white arrows heads).

Abdominal organs and GI tract are common targets for mast cell infiltration. GI tract symptoms are also thought to be related to mast cell infiltration of lamina propria and mediator release. Although it is rare, malabsorption may occur as a result of mast cells’ proinflammatory effect on the small bowel. Therefore, thickened walls of small intestine, large intestine, and stomach are common radiological features in SM. They also can have nodular appearance mainly due to lamina propria infiltration [28]. On CT scan, the bowel wall thickening is visualized and can be associated with strictures and lymph node enlargement (Figure 3) [29]. Esophagus, duodenum, and colon are also involved in SM with similar manifestations. Mast cells effect on esophagus may show esophageal varices, strictures and inflammation [29]. Significant histamine release from mast cells can lead to increasing gastric secretion and peptic ulcer disease of the stomach and duodenum. Colonic manifestations are rare in SM, they may include mucosal nodules, edema, and polypoid lesions [30].

#### 6.2.2. Liver and Spleen

**Figure 4:**
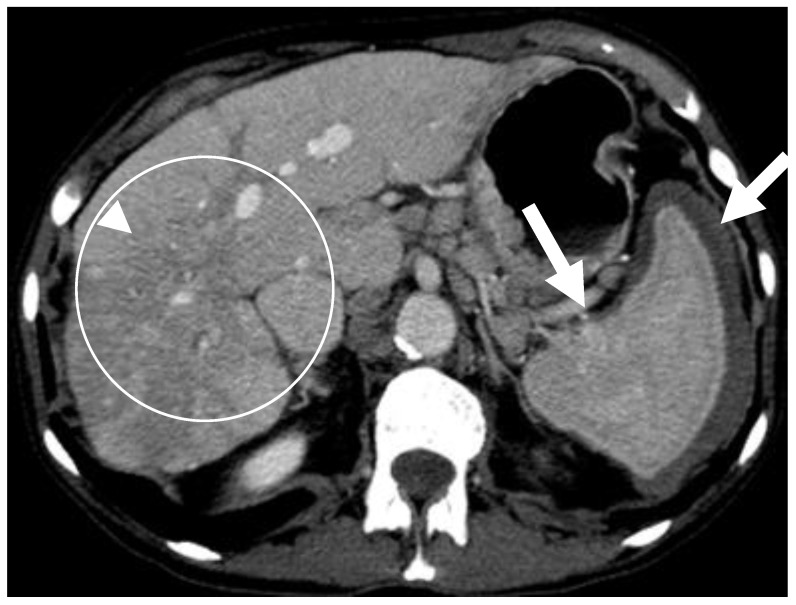
A 59-year-old lady with a 20-year history of systemic mastocytosis. Axial contrast-enhanced CT demonstrates hepatomegaly and a large heterogeneous mass with areas of arterial enhancement and heterogeneous hypoattenuating apperance on portal venous phase (circled), increased retraction of the hepatic capsule related to volume loss (arrowhead), and an abnormal heterogenous appearance of the spleen with surronding ring of hypoattenuating soft tissue (white arrows).

**Figure 5:**
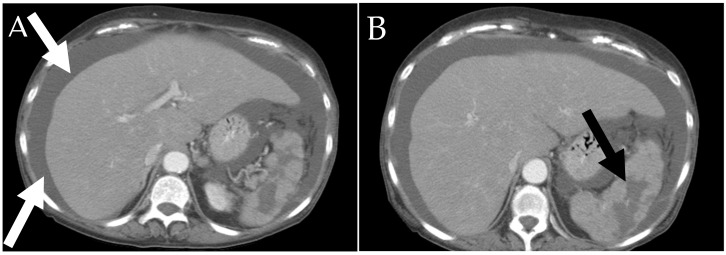
A 69-year-old female with history of systemic mastocytosis. (**A**,**B**) Axial contrast enhanced CT shows ascites (white arrows) which appears as free fluid at the liver margin. An enlarged heterogeneous spleen with lobulated contour and evindence of infarction (black arrow).

The liver is affected in 61% of SM cases, especially in cases of aggressive variants [12]. Hepatomegaly with ascites and periportal adenopathy are the most common abdominal imaging findings in SM (Figure 3) [31]. In early SM, hepatic involvement may remain asymptomatic. Yet, hepatic complications in SM occur due to mast cells obstructing the intrahepatic ducts causing portal hypertension leading to ascites development. Other cause of ascites may include the release of angiogenetic mediators from the mast cells [32]. Early imaging is valuable in detecting enlarged liver and spleen sizes as it can be missed by physical examination [28]. CT is useful to identify the typical features but is perhaps more useful in excluding other disease processes (Figure 3 and Figure 4). Splenomegaly is also a common feature and is present in 61% of SM cases [31]. It is believed that mast cell infiltration is the main cause of organomegaly [28]. The spleen appears heterogeneous on CT and may be associated with infarctions which appear as hypoattenuating wedge shaped regions. This may result in lobulated splenic contour (Figure 5).

### 6.3. Future Implications

Recently, it has suggested that mast cells (MCs), microRNAs (miRNAs), Kirsten rat sarcoma (KRAS) and v-raf murine sarcoma viral oncogene homologue B (BRAF) plat a role in cancer growth and progression. Various effort has been exerted to evaluate their correlation with colorectal cancer aiming to improve patient survival and quality of life. They can act what so-called ‘ideal biomarker ‘which are non-invasive, cost effective and easily measured biomarkers which can used for colorectal cancer screening [33].

### 6.4. Lymphatic

**Figure 6:**
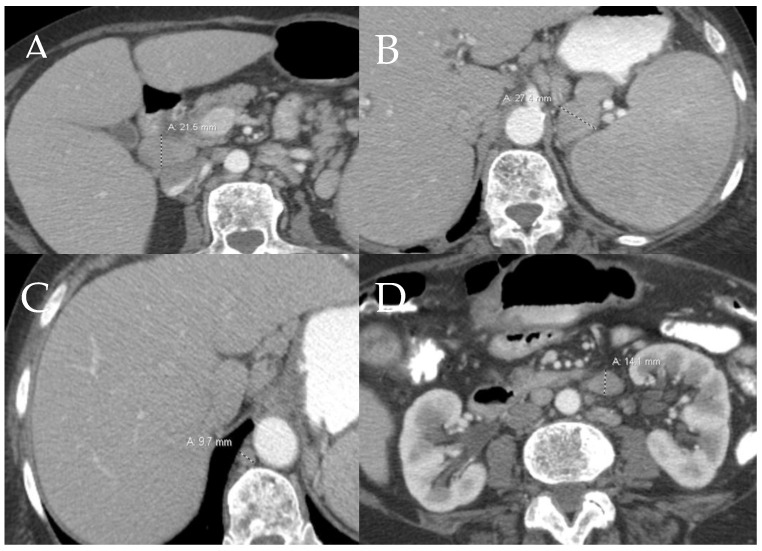
An 80-year-old female with history of aggressive systemic mastocytosis. (**A**–**D**) Axial contrast-enhanced CT abdomen shows multi-compartmental lymphadenopathy.

**Figure 7:**
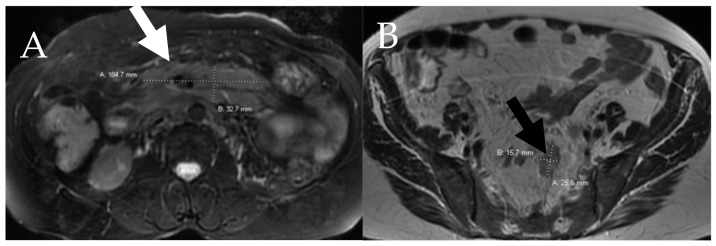
A 60-year-old female with aggressive systemic mastocytosis. (**A**) Axial fat-saturated T2W image through the central abdomen shows a large confluent nodal mass in the central mesentery (white arrow). (**B**) Axial T2W through the pelvis shows superior hemorrhoidal lymphadenopathy (black arrow).

Lymphadenopathy is common in patients with SM due to mast cell tendency to infiltrate lymph node paracortex and follicles. It is mainly detected in SM associated with hematologic neoplasm. Peripheral adenopathy is present in 26% of cases and central adenopathy is present in 19% of cases [12]. The most common lymphadenopathy locations include periportal, retroperitoneal, mesenteric, and axillary regions [31]. CT and ultrasound can help identify involved lymph node groups. Both discrete enlarged lymph nodes and large nodal conglomerates can be seen; however, the appearance is not specific to mastocytosis (Figure 6 and Figure 7) [34].

### 6.5. Skin

**Figure 8:**
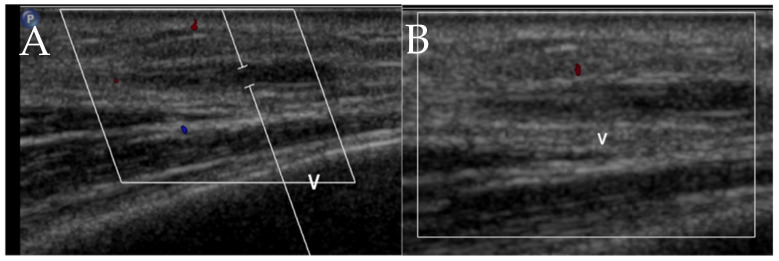
A 60-year-old female with history of systemic mastocytosis combined with cutaneous mastocytosis (**A**,**B**) Superficial thrombophlebitis in the right forearm.

**Figure 9:**
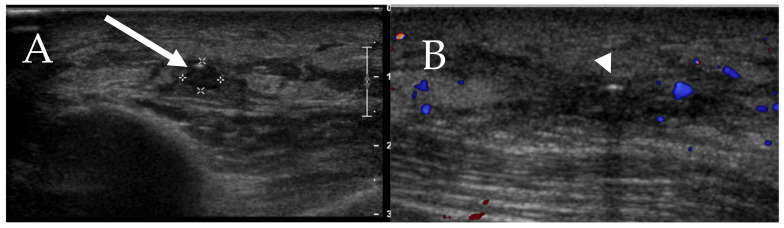
A 71-year-old male with systemic mastocytosis. Doppler ultrasound of the right anterior upper chest wall shows (**A**) heterogeneous hypoechoic area due to mild subcutaneous edema that probably represents a focal of inflammatory process (arrow). (**B**) Punctate echogenicity with posterior acoustic shadowing in the area, may represent a small calcification or a focus of air (arrowhead).

Cutaneous manifestations of mastocytosis are better evaluated on dermatologic exam and histopathology. These changes are due to focal inflammatory process related to mast cell infiltration of the skin [16]. Radiographically, mild cutaneous or subcutaneous edema can be seen in a setting of superficial thrombophlebitis which can occur in cases of SM (Figure 8). Typical sonographic findings include dilated non-compressible superficial subcutaneous vein with absent color Doppler signal and mild edema/small amount of fluid in the subcutaneous tissues (Figure 9) [35]. Punctate intraluminal echogenic foci may be present due to small calcifications with chronic thrombosis.

### 6.6. Central Nervous System

**Figure 10:**
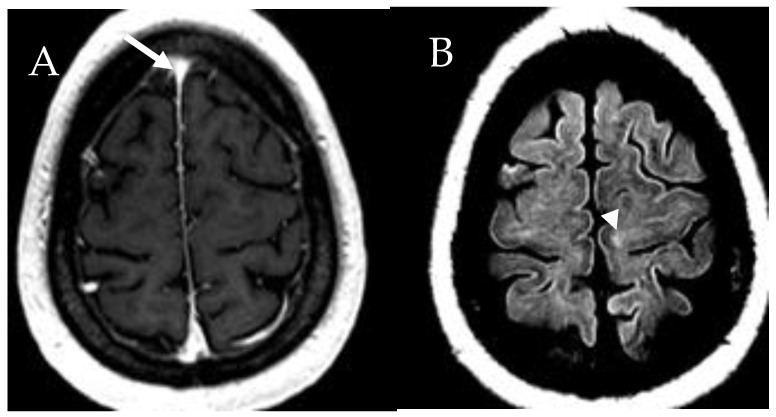
A 55-year-old male patient with systemic mastocytosis. (**A**) Axial postcontrast T1-weighted image demonstrates punctate focus of enhancement within the right frontal lobe cortex (arrow). (**B**) Focal T2 hyperintensity in the subcortical left temporal lobe (arrowhead).

Neuropsychiatric abnormalities have been reported in select number of patients with mastocytosis. Hence, evaluation of the brain is important in both CM and SM cases. MRI is usually used to detect brain abnormalities in mastocytosis. It shows punctuate asymmetric homogeneously enhancing lesions in the cortical, subcortical, or deep regions of the brain (Figure 10) [36]. According to Boddaert et al., white matter abnormalities and increased perfusion to the basal ganglia were detected in 49% of the study cases [36]. However, MRI findings are non-specific, and their appearance is thought to be related to an inflammatory reaction in patients with mastocytosis.

### 6.7. Cardiac and Respiratory

**Figure 11:**
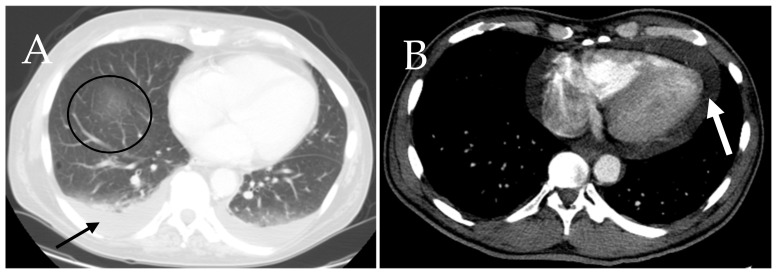
A 64-year-old male with mastocytosis. Axial contrast-enhanced CT scan of thorax demonstrates (**A**) areas of fluid at the base of the lung denoting pleural effusion (black arrow), (**B**) fluid surrounding the heart as an evidence of pericardial effusion (white arrow) and focal pulmonary ground glass opacity (circle).

Cardiac and pulmonary features in mastocytosis are rare [28]. Lung involvement in mastocystosis is believed to be triggered mainly by the fibrotic and proinflammatory effects of mast cells [28]. Pulmonary abnormalities described include scattered areas of fibrosis, bilateral interstitial fibrosis, cystic changes and multiple pulmonary nodules [24,37]. The main cardiovascular manifestation associated with SM is atherosclerotic plaque formation [38]. Thoracic CT imaging can help detect changes that occur during disease progression. CT findings include pericardial effusion, pleural effusions, pulmonary fibrosis, cystic lung lesions, and pulmonary nodules (Figure 11). CT also may show mediastinal and hilar lymphadenopathy [39].

### 6.8. Coronavirus and Mastocytosis

As we are in the midst of the COVID-19 pandemic, it is important to discuss the relationship of coronavirus infection and mastocytosis. SARS-CoV-2 is linked to high mortality and morbidity secondary to massive release of pro-inflammatory cytokines and thrombogenic mediators, leading to lung damage. It has been reported that significant number of children who tested positive for SARS-CoV-2 and recovered or had mild symptoms developed diffuse multi-organ symptoms months after the infection called Multisystem Inflammatory Syndrome (MIS-C).

The U.S. Centers for Disease Control has also described the detection of a similar condition in adults called Multisystem Inflammatory Syndrome (MIS-A). These conditions are identified by similar symptoms related to Mast Cell Activation Syndrome (MCAS, US ICD-110 code D89.42-idiopathic mast cell activation syndrome). Accordingly, the possibility of MCAS has to be considered in any patient with MIS and/or multisystem inflammatory symptoms. In both cases, these syndromes should be tackled with liposomal formulation (in olive pomace oil) of the flavone luteolin (e.g., PureLut^®^ or FibroProtek^®^) along with the antihistamine rupatadine, which also exhibit an anti-platelet activating factor (PAF) activity and inhibit mast cells that have been involved in the pathogenesis of cytokine storms in COVID-19 [40].

It was also proposed that coronavirus infection can be inhibited by anti-inflammatory cytokines related to the IL-1 family members. As coronavirus infection is principally attacked by immune cells comprising of masts cells in the submucosa of the respiratory tract and act as blockade for protection against microorganisms. Early inflammatory chemical complexes are released when virus activate the mast cells, however late activation trigger release of pro-inflammatory IL-1 family members including IL-1, IL-6 and IL-33 [41].

## 7. Conclusions

The diagnosis of mastocytosis is challenging and relies on physical examination, histopathological findings, genomic testing, and laboratory results. Making this diagnosis can be difficult due to rarity of this disorder, non-specific symptoms and imaging findings. Imaging plays a central role in detection of many non-specific findings seen in mastocytosis. Moreover, imaging can help characterize patients presenting with SM as either aggressive or indolent SM. An understanding of mastocytosis pathology and clinical presentations along with the relevant imaging findings can aid in early diagnosis and optimal patient management.

## Figures and Tables

**Table 1:** B and C findings of systemic mastocytosis.

B Findings	C Findings
1. Mast cells Infiltration in BM > 30% and/or serum tryptase level > 200 ng/mL. 2. Dysmyelopoiesis with normal or slightly abnormal blood cell count. 3. Organomegaly (without impaired organ function) as the following: Palpable hepatomegaly without liver impairment Palpable lymphadenopathy or size of the visceral lymph nodes > 2 cm on CT scan Palpable splenomegaly	1. Cytopenias: Absolute neutrophil count < 1000 g/dL Hemoglobin < 10 g/dL Platelet < 100,000 μL 2. Organomegaly with impaired organ function as the following: Palpable hepatomegaly with ascites Palpable splenomegaly with hypersplenism 3. GI tract: Malabsorption resulting in weight loss and low albumin level. 4. Skeletal system: osteolysis that may be associated with osteoporosis and pathologic fractures

**Table 2:** Major and minor WHO criteria for systemic mastocytosis.

Major Criteria	Minor Criteria
Mast cells infiltrate the bone marrow in different foci (i.e., >15 mast cell collected in one location in bone marrow biopsy and/or other extra-cutaneous organs).	(a) Atypical mast cells on bone marrow smears (25%) or spindle shaped infiltrates on other organs. (b) D816V point mutation of KIT gene detected in the bone marrow or other organs. (c) CD 2/CD25 detected on mast cells in bone marrow or other extracutaneous organs. (d) Serum tryptase level > 20 ng/mL.
